# Genome characterization of a *Eucalyptus* natural mutant

**DOI:** 10.1186/1753-6561-5-S7-P65

**Published:** 2011-09-13

**Authors:** Maria CP Fuchs, Juliana C Lourenção, Evandro V Tambarussi, Tânia M Bortoloto, Shinitiro Oda, Fábio TS Nogueira, Celso L Marino

**Affiliations:** 1 Departamento de Genética, Instituto de Biociências, Universidade Estadual Paulista (UNESP), Distrito de Rubião Jr s/n CEP 18618-000, Botucatu (SP), Brazil; 2 Departamento de Genética, Escola Superior de Agricultura “Luiz de Queiroz” (ESALQ), Universidade de São Paulo (USP), Avenida Pádua Dias, 11 CEP 13418-900, Piracicaba (SP), Brazil; 3 Empresa Suzano Papel e Celulose SA, Av. Dr. José Lembo, 1010, CEP 18207-780, Itapetininga (SP), Brazil

## Background

**Figure 1:**
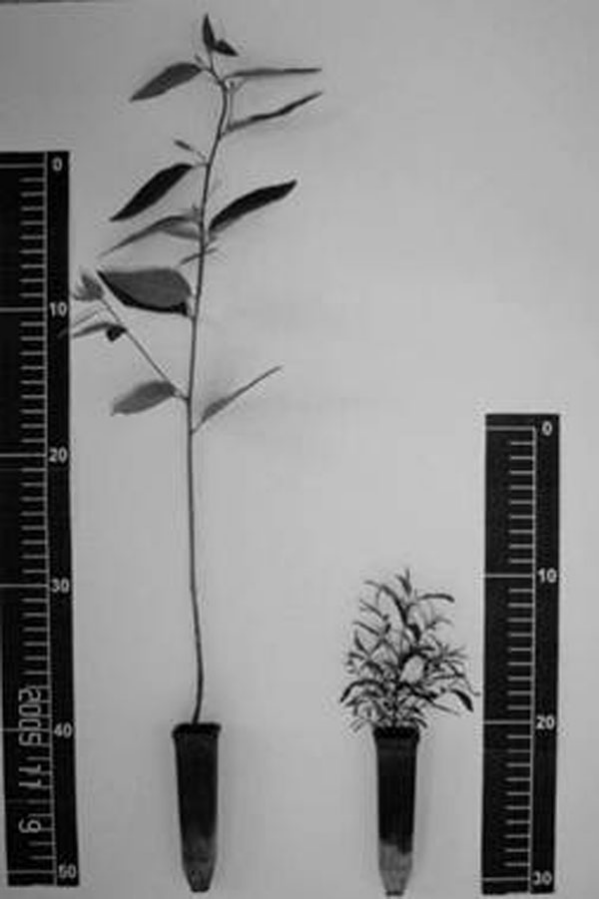
Normal (left) and abnormal (right) seedlings.

*Eucalyptus* genetic improvement is time-consuming mainly because its long time to reach reproductive maturity along with its mixed mating system [6]. As a result, the production of pure lines as in maize is impracticable. The heterozygous status hides deleterious recessive genes that, when in homozygosity can reduce seedling survival due to physiological changes, resulting in considerable loss in seedling production [6]. Therefore, it would be important to identify the gene(s) associated with inbreeding depression. There are very few studies involving the characterization of deleterious recessive genes in Eucalyptus. We detected an anomaly in the offspring obtained from a controlled cross of *Eucalyptus grandis*. The anomaly appears in a ratio of one abnormal to three normal seedlings, suggestive of a Mendelian segregation. Based on the segregation ratio, we hypothesized that the character is controlled by a single gene, with a homozygous genotype for the recessive allele showing abnormal seedlings. The parents showed normal phenotype and they have no relationship, excluding the possibility of inbreeding depression due to identity by descent. The abnormal seedlings die in a few months and show different characters: high shoot branching, height reduction, leaf area reduction, and changes in leaf shape (Figure 1). In this scenario, we aimed to characterize the genomic and genetic causes of the observed anomaly.

## Methods

The full-sib individuals generated at Suzano Papel and Celulose SA company were divided into normal and abnormal seedlings. The ratio between normal and abnormal seedlings was tested by chi-square analysis. DNA was extracted according to Ferreira and Grattapaglia [1]. The DNA concentration was quantified using a spectrophotometer (Thermo Scientific NanoDrop™ 1000), and subjected to electrophoresis in 1% agarose gel and stained with ethidium bromide. DNA from ten normal individuals were randomly selected and mixed, creating the normal bulk, and the same was performed to create the abnormal bulk. The bulks were amplified with random primers (kits AD, AE, AF, AJ, and AK – Operon Technologies, Inc.). Amplicons were submitted to electrophoresis in 1% agarose gel stained with ethidium bromide. Polymorphic markers detected by RAPD were converted to SCAR (Sequence Characterized Amplified Regions) markers. DNA fragments from polymorphic marker were purified from agarose gel using the illustra GFX PCR DNA (GE Healthcare), cloned into vector pGEM-T Easy (Promega) and inserted into UltraMAX DH5α-FT competent *Escherichia coli* cells (Life Technologies). DNA was sequenced in an ABI3100 Genetic Analyzer (Applied Biosystems). SCAR primers were designed based upon the above mentioned sequencing and further used to amplify DNA fragments from bulks and six individuals: three anomalous, one individual from normal bulk; and two normal individuals of unrelated populations (BAC and Brasuz). Amplicons of the six individuals were purified, cloned into vector pGEM-T Easy (Promega) and inserted into DH5αcompetent *E. coli* cells (Life Technologies). Six colonies from each individual were amplified with the SCAR primerss. Purified product of amplification was sequenced by ABI3100 Genetic Analyzer (Applied Biosystems). SCAR derivedsequences were compared among themselves and with the eucalypt genome (Phytozome v7.0 database - http://www.phytozome.net/) using BLAST. The genomic region identified was analyzed by FGENESH tool (Softberry, Inc. – http://www.softberry.com) to find predicted genes in the region. Predicted genes were translated by EXPASY translate tool (http://expasy.org), and the protein analyzed by PFAM database (http://pfam.sanger.ac.uk) for domain identification.

## Results

Chi-square analysis confirmed Mendelian segregation of 3 normal: 1 anomalous seedlings in the tested progeny. The data indicate that the anomaly is a monogenic character that manifests when the deleterious recessive allele is in homozygosity. Among the random primers tested only one showed polymorphism with the segregation pattern. This marker appears in all abnormal and in 31% of normal individuals. The RAPD marker was converted into a SCAR marker and its segregation pattern confirmed. All abnormal individuals and 22.2% of normal individuals showed the marker. Sequencing of the SCAR marker was done to identify the possible genomic region where it is linked and detected a polymorphism of two base pair between anomalous (mutant) and normal individuals. Our *in silico* analysis showed the polymorphism in an intergenic region, localized between two protein-coding with a Bet v1-type domain belonging to the PR10 family (Pathogenesis-related protein 10). In general, this is associated with plant defense function in response to biotic and abiotic stresses [3-5]. However, there are reports that PR10 has important roles in biological processes [4,5].

## Conclusions

According to our results, we propose that the anomalous character might be caused by a major effect gene. The marker found is related with the recessive; therefore, normal individuals with the marker are likely heterozygous. The polymorphism detected in the SCAR sequence, suggests that the mutation might be related to the two base substitution observed. However, at this point, it is unclear whether these base substitutions are involved affecting close genes (such as the identified PR10 proteins) or any other unknown genomic region. Expression analysis of the PR10-coding genes might provide clues to answer this biological question.
